# Factors influencing the cost-effectiveness of novel oral anticoagulants compared to vitamin K antagonists in patients with atrial fibrillation: a systematic review

**DOI:** 10.3389/fphar.2025.1441754

**Published:** 2025-03-28

**Authors:** Yan Li, Xintian Wang, Aixia Ma, Pingyu Chen, Hongchao Li

**Affiliations:** ^1^ School of International Pharmaceutical Business, China Pharmaceutical University, Nanjing, China; ^2^ Center for Pharmacoeconomics and Outcomes Research, China Pharmaceutical University, Nanjing, China

**Keywords:** atrial fibrillation, novel oral anticoagulants, vitamin K antagonists, cost-effectiveness, systematic review

## Abstract

**Background:**

Atrial fibrillation (AF) is a common clinical arrhythmia, primarily associated with the risk of stroke and various thromboembolic events, imposing significant clinical and economic burdens on patients and societies. This study aimed to review the relevant pharmacoeconomic evaluations of novel oral anticoagulants (NOACs) compared to vitamin K antagonists (VKAs) in patients with AF and explore the influencing factors and general trends of economic evaluations.

**Methods:**

This review qualitatively analyzed the basic characteristics, model structure, and basic results of all included studies. Moreover, a cross-sectional and longitudinal comparative analysis of costs, health outcomes, and cost-effectiveness results of studies in the United States, China, and the United Kingdom was conducted. Additionally, this study employed multivariate binary logistic regression to explore the influencing factors and general trends of the cost-effectiveness between NOACs and VKAs across all included studies.

**Results:**

A total of 103 studies were included, comprising 218 comparisons between NOACs and VKAs. Total costs and health outcomes measured in studies with different countries and baseline characteristics exhibited considerable variations. However, NOACs generally had higher total costs than VKAs and resulted in more health outcomes for patients. The binary logistic regression analysis revealed that the country’s economic development level, study perspective, and cycle length significantly influenced cost-effectiveness results.

**Conclusion:**

In high-income countries, NOACs are generally considered cost-effective, while VKAs may remain an attractive strategy in middle- and low-income countries. Additionally, factors such as drug prices, patient baseline characteristics, and model settings could impact the costs, health outcomes, and cost-effectiveness results of studies. Conducting relevant pharmacoeconomic research based on specific populations and study contexts is essential.

## Highlights

• Drug prices of NOACs are a critical factor in total treatment costs and can significantly impact cost-effectiveness results. China’s centralized drug procurement policy has lowered NOACs prices and improved their cost-effectiveness.
• The patients’ baseline characteristics, study contexts, and model settings influence the cost, health outcomes, and cost-effectiveness results of NOACs and VKAs. Conducting relevant pharmacoeconomic evaluations based on specific populations and study contexts is essential.
• Compared to VKAs strategies, NOACs strategies generally lead to greater health benefits and higher costs. The country’s economic development level, study perspective, and cycle length significantly influenced cost-effectiveness results.

## 1 Introduction

Atrial fibrillation (AF) is a common clinical arrhythmia, primarily associated with the risk of stroke and various thromboembolic events. Thromboembolic complications are mainly related to changes in systemic blood flow dynamics in AF patients, particularly stroke, which carries a high mortality and disability rate, significantly impacting patients’ quality of life (Hindricks et al., 2021). Epidemiological data indicate that in 2019, there were approximately 59.7 million AF patients globally (including atrial flutter). In high-income countries (HICs), the burden of AF is escalating due to population aging and the presence of risk factors such as hypertension and diabetes (Camm et al., 2010). In low- and middle-income countries (LMICs), while the risk from age-related factors may be less pronounced, risk factors like hypertension and diabetes are often underdiagnosed and inadequately controlled, suggesting that the future burden of AF may increase further (Agbor et al., 2019). Studies project that the incidence and prevalence of AF are expected to continue rising in the next 30 years, making it one of the most significant global epidemiological and public health challenges (Lippi et al., 2021).

Currently, common anticoagulant medications used for stroke prevention in AF patients include vitamin K antagonists (VKAs), represented by warfarin, and novel oral anticoagulants (NOACs), which mainly consist of direct thrombin inhibitors like dabigatran and factor Xa inhibitors like apixaban, rivaroxaban, and edoxaban. Relevant clinical studies (Giugliano et al., 2013; Connolly et al., 2009; Granger et al., 2011; Patel et al., 2011) have shown that NOACs are non-inferior to VKAs in preventing stroke or systemic embolism, and have the advantage of significantly reducing bleeding risks while maintaining anticoagulation efficacy. NOACs are increasingly becoming a new trend in anticoagulant therapy for AF, attributed to their ease of use, no need for international normalized ratio (INR) monitoring, better compliance, and fewer food-drug interactions (Steffel et al., 2021). However, the relatively high cost of NOACs has limited their widespread use to some extent.

In terms of cost-effectiveness, the relatively higher cost of NOACs restricts their use in resource-limited LMICs, where VKAs remain an attractive strategy. Whereas in HICs, NOACs seem to be the new conventional strategy (Noviyani et al., 2022). Furthermore, as NOACs have been on the market for some time, the costs of these drugs have changed in some countries and regions, exerting a significant influence on cost-effectiveness results.

Pharmacoeconomic evaluation can provide evidence to help decision-makers optimize the utilization of scarce healthcare resources. This study aimed to systematically review existing pharmacoeconomic evaluations of NOACs compared to VKAs in patients with AF, analyze relevant studies from different countries and years, and explore the influencing factors of the cost-effectiveness, to provide valuable references for future pharmacoeconomic research and healthcare decision-making.

## 2 Methods

### 2.1 Eligibility criteria

This systematic review focused on original research studies on the pharmacoeconomic evaluation of various AF management strategies. To be included, studies had to adopt a cost-effectiveness analysis (CEA) or cost-utility analysis (CUA) to assess the cost-effectiveness of NOACs (apixaban, edoxaban, apixaban, and dabigatran) compared to VKAs (warfarin) in patients with AF. The reported outcome measures included costs, effectiveness, incremental cost-effectiveness ratio (ICER), and quality-adjusted life years (QALYs). Only studies published in English or Chinese language and involving human participants were included. Duplicate publications, case reports, conference abstracts, lectures, reviews, and studies with inaccessible full texts were excluded. Additionally, studies with incomplete information required for pharmacoeconomic evaluation, such as studies of disease burden or cost measurement, or budget impact analysis, were also excluded.

### 2.2 Search strategy

A comprehensive and systematic search was performed in PubMed, Embase, Web of Science (WOS), The Cochrane Library, China National Knowledge Infrastructure (CNKI), VIP Database for Chinese Technical Periodicals (VIP), and Wanfang Data from the inception of the databases to November 2022. A combination of subject headings and free-text terms was used for the search, tailored according to each database’s characteristics and requirements. Search terms included “atrial fibrillation,” “vitamin K antagonist,” “oral anticoagulant,” “economic evaluation,” “pharmacoeconomic,” “cost-effectiveness,” and “cost-utility.” The detailed search strategies for each database are provided in the Supplementary Materials.

### 2.3 Data extraction and analysis

Two reviewers independently screened the literature, extracted data, and conducted quality assessments of included studies using a standardized data extraction form to ensure consistency and accuracy. In cases of disagreement or disputes, discrepancies were resolved through discussion, and if necessary, a third reviewer was consulted for confirmation. Initial screening was based on titles and abstracts, while final inclusion was based on the full-text article. The information extracted included: (1) basic information about the included studies (title, first author, publication year, country of the study, target population, interventions, etc.); (2) model structure and study assumptions (model type, time horizon, cycle length, etc.); (3) key analysis results (costs, health outcomes, cost-effectiveness results, study conclusions, etc.).

### 2.4 Quality assessment

The Consolidated Health Economic Evaluation Reporting Standards 2022 (CHEERS 2022) statement (Husereau et al., 2022) was employed to assess the reporting quality of the included studies. Additionally, the literature quality classification standards proposed by Degeling (Degeling et al., 2020) were referenced, defining studies with a compliance rate of >80% as high quality, 60%–80% as moderate quality, and <60% as low quality.

### 2.5 Data processing and statistical analysis

This review qualitatively analyzed the basic characteristics, model structure, study assumptions, and basic analysis results of all included studies. Moreover, a cross-sectional and longitudinal comparative analysis of cost and health outcomes of studies in the United States (US), China, and the United Kingdom (UK) was conducted. The main reason for focusing on these three countries was because they had a sufficient number of studies, which allowed this review to observe trends and conduct comparisons between different countries.

Additionally, a multivariate binary logistic regression analysis was performed to explore the influencing factors and general trends of cost-effectiveness results between NOACs and VKAs across all included studies from all countries. The dependent variable was defined as cost-effectiveness results (1 = NOACs are cost-effective compared to VKAs; 0 = NOACs are not cost-effective compared to VKAs). Independent variables, including study perspective, cycle length, country’s economic development level, funding status, and other relevant factors, were selected based on prior literature and clinical relevance. To assess potential multicollinearity, variance inflation factor (VIF) analysis was performed, confirming that all values were below the threshold of 5. The study utilized Excel 2016 for data extraction and processing, and SPSS 26.0 for regression analysis.

## 3 Results

**FIGURE 1:**
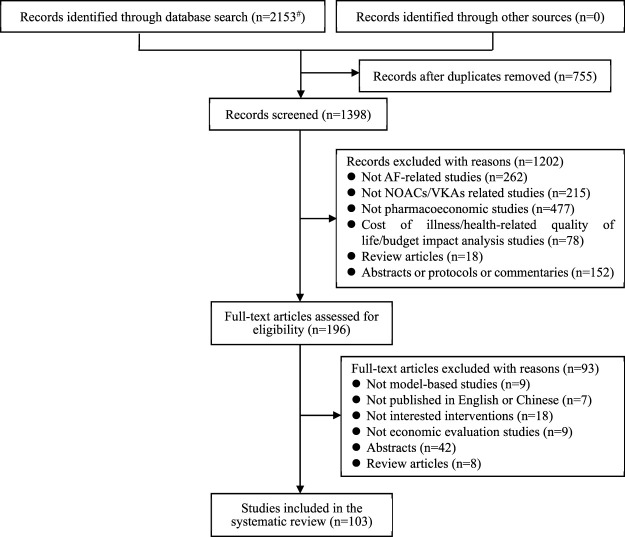
Flow of study selection through different phases of the systematic review according to the PRISMA statement [#Detailed search results: PubMed Database (n = 271), Web of Science Database (n = 1,098), The Cochrane Library Database (n = 88), Embase Database (n = 525), CNKI (n = 38), VIP (n = 28), Wanfang Data (n = 105)].

A total of 2,153 records were initially identified using the search strategies. After the screening process, 103 studies (Wu et al., 2021; Wang et al., 2020; Salcedo et al., 2019; Hospodar et al., 2018; Altawalbeh et al., 2018; Vargas et al., 2018; Hernandez et al., 2017; Shah et al., 2016; Nguyen et al., 2016; Salata et al., 2016; Magnuson et al., 2015; Clemens et al., 2014; You, 2014; Harrington et al., 2013; Canestaro et al., 2013; Lee et al., 2012a; Lee et al., 2012b; Kamel et al., 2012a; Shah and Gage, 2011; Zhou et al., 2022; Yang, 2021; Wei et al., 2021; Liu et al., 2021; Wang et al., 2022; Cui et al., 2020; Lv and Yang, 2019; Huang et al., 2019; Wu et al., 2016a; Wu et al., 2016b; Chen and Han, 2016; Wan et al., 2014; Wu et al., 2014; Bowrin et al., 2020a; Thom et al., 2019; Zheng et al., 2014; Dorian et al., 2014; Pink et al., 2014; Kansal et al., 2012; Kamel et al., 2012b; Raunbak et al., 2022; Escobar Cervantes et al., 2022; Choi et al., 2022; Aghoram et al., 2022); (Mendoza et al., 2019; Kim et al., 2019; Hersi et al., 2019; Dwiprahasto et al., 2019; Lekuona et al., 2019; van Hulst et al., 2018; Vilain et al., 2017; López-López et al., 2017; Liu and Chen, 2017; Lanas et al., 2017; Athanasakis et al., 2017; Zhao et al., 2016; Li et al., 2016; Hallinen et al., 2016; Pepe et al., 2015; Kamae et al., 2015; Giorgi et al., 2015; Carles et al., 2015; Rognoni et al., 2015; Mensch et al., 2015; Krejczy et al., 2015; Kongnakorn et al., 2015; Janzic and Kos, 2015; Barón et al., 2015; Wisløff et al., 2014; Wang et al., 2014; Verhoef et al., 2014; Stevanovic et al., 2014; Rognoni et al., 2014; Morais et al., 2014; Lanitis et al., 2014; Kourlaba et al., 2014; Jarungsuccess and Taerakun, 2014; Chevalier et al., 2014; Ademi et al., 2015; Krejczy et al., 2014; Chang et al., 2014; Wouters et al., 2013; Pletscher et al., 2013; Nshimyumukiza et al., 2013; Kleintjens et al., 2013; Andrikopoulos et al., 2013; Bowrin et al., 2020b; Coyle et al., 2013; de Jong et al., 2019; de Pouvourville et al., 2020; Hallinen et al., 2021; Hori etal., 2020; Kansal etal., 2017; Langkilde et al., 2012; Liao et al., 2020; Lorenzoni et al., 2021; Ng et al., 2020; Rivolo et al., 2021; Walter et al., 2021; Dilokthornsakul et al., 2020; Rattanachotphanit et al., 2019; Davidson et al., 2013; Sorensen et al., 2011; Freeman et al., 2011) were finally included in this systematic review, of which 9 were in Chinese and 94 were in English. The literature screening process is presented in Figure 1, following the PRISMA statement (Moher et al., 2009).

### 3.1 Quality assessment results

The compliance rate of the included studies according to the CHEERS 2022 checklist ranged from 57.1% to 92.9%, with an average compliance rate of 77.9%. Among them, 37 studies (35.9%) were classified as high quality, with a compliance rate above 80%; 64 studies (62.1%) were classified as relatively high quality, and two studies (1.9%) (Wang et al., 2022; Huang et al., 2019) published in Chinese journals had a compliance rate less than 60% and were considered low quality. Overall, the quality of the included literature was relatively high. A summary figure of the quality assessment is provided in the Supplementary Materials.

### 3.2 Basic characteristics of included studies

The 103 included studies were conducted in 32 different countries, including the US, China, the UK, and others. Among them, 43 studies (41.7%) were conducted from the perspective of the healthcare system, 37 studies (35.9%) from the perspective of the payer, and 22 studies (21.4%) from the societal perspective. Twenty-nine studies (28.1%) were not funded, while the remaining studies received funding from industry, research institutions, or government sources. The basic characteristics of the included studies are shown in Table 1.

**TABLE 1:** Characteristics and methods of included studies.

Category	n (%) (N = 103)	Category	n (%) (N = 103)
Country		Time horizon	
US	19 (18.4%)	5 years	1 (1.0%)
China	13 (12.6%)	10 years	1 (1.0%)
United Kingdom	7 (6.8%)	Lifetime	101 (98.0%)
Spain	5 (4.9%)		
Others (Canada/France/Thailand/…/n < 5)	59 (57.3%)		
Perspective		Cycle length	
Healthcare system	43 (41.7%)	-	3 (2.9%)
Payer	37 (35.9%)	<3 months	40 (38.8%)
Societal	22 (21.4%)	3 months	40 (38.8%)
Patient	1 (1.0%)	1 year	20 (19.4%)
Evaluation method		Cost identification	
CEA, CUA	25 (24.3%)	Only DMCs	87 (84.5%)
CUA	78 (75.7%)	DMCs, DNMCs	5 (4.9%)
		DMCs, DNMCs, ICs	9 (8.7%)
		DMCs, ICs	2 (1.9%)
Funding		Half-cycle correction	
Industry	45 (43.7%)	N	93 (90.3%)
Government/Scientific research institute	29 (28.1%)	Y	10 (9.7%)
N	29 (28.1%)		
Model type		Model validation	
Markov cohort	98 (95.1%)	N	92 (89.3%)
Micro-simulation Markov	4 (3.9%)	Y	11 (10.7%)
DES	1 (1.0%)		

“-“, not report; Y, yes; N, no; CEA, cost-effectiveness analysis; CUA, cost-utility analysis; DES, discrete events simulation model; DMCs, direct medical costs; DNMCs, direct non-medical costs; ICs, indirect costs.

### 3.3 Model structure and study assumptions

Among the included studies, only one study used the discrete events simulation model (DES) (Pink et al., 2014), while the rest used Markov models, including the Markov cohort model and micro-simulation Markov model. The model structures for the treatment of AF with anticoagulants were relatively complex, with the number of health states ranging from 4 (Carles et al., 2015) to 29 (Sorensen et al., 2011), mainly beginning with the stable state with AF, followed by multiple cardiovascular and cerebrovascular event-related states and death. Among the included studies, 101 studies (98.0%) used a lifetime horizon to evaluate the cost-effectiveness of anticoagulant therapy for AF.

The cycle length of models should be sufficiently short to accurately simulate the frequency of relevant events. Among the included studies, 20 studies (19.4%) used a one-year cycle, 40 studies (38.8%) used a 3-month cycle, and another 40 studies (38.8%) used a cycle of less than 3 months, including 1 month, 6 weeks, and 2 weeks. In addition, the discretization process of the Markov model typically results in errors, which can be mitigated through half-cycle correction. Among the 103 included studies, only 10 studies (9.7%) reported the use of the correction method.

### 3.4 Costs and health outcomes

In terms of cost composition, all studies included direct medical costs, while a subset of studies adopting a societal perspective additionally considered direct non-medical costs (n = 14) and indirect costs (n = 11). In regards to health outcome measure, all included studies adopted the CUA approach, using QALYs as the health outcome measure. Additionally, 25 studies (24.3%) also used the CEA approach, using life years (LYs) as the health outcome measure.

**FIGURE 2:**
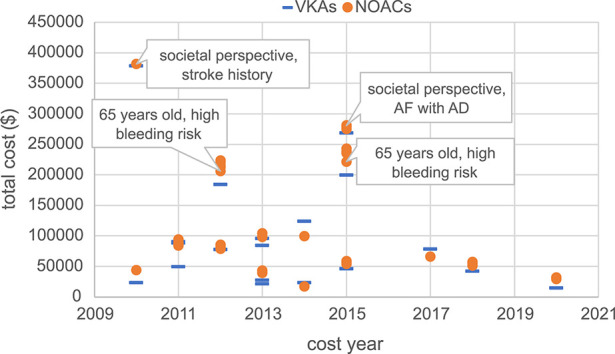
Total costs of studies in the US.

**FIGURE 3:**
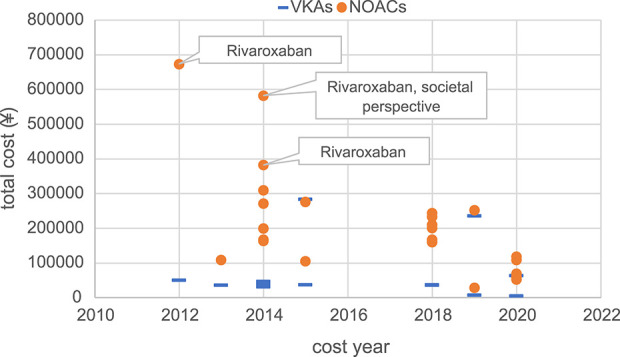
Total costs of studies in China.

**FIGURE 4:**
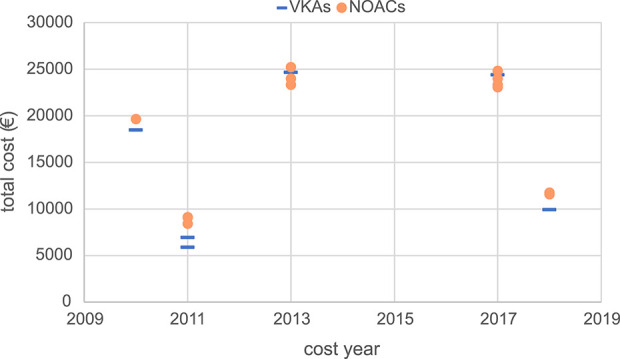
Total costs of studies in the UK.

**FIGURE 5:**
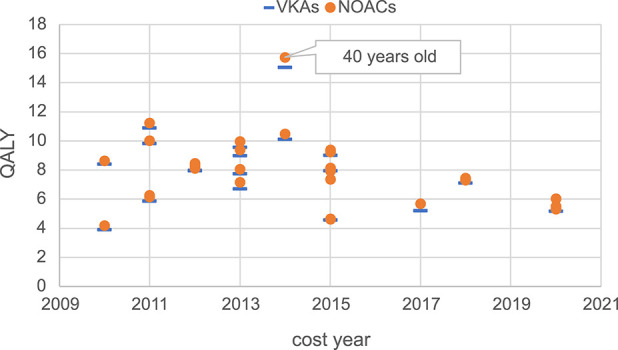
Total health outcomes of studies in the US.

**FIGURE 6:**
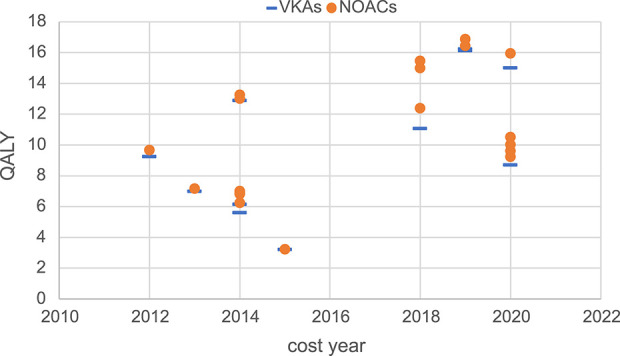
Total health outcomes of studies in China.

**FIGURE 7:**
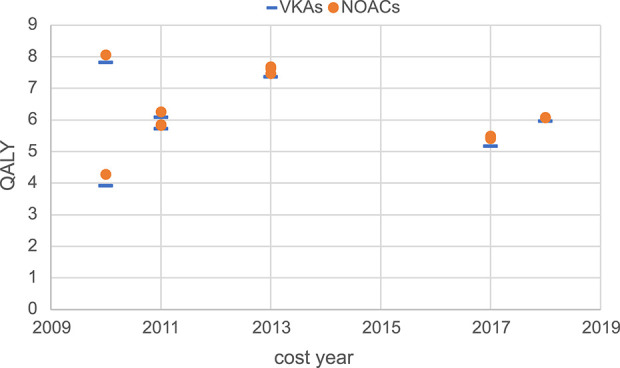
Total health outcomes of studies in the UK.

Moreover, this study conducted a summary analysis of the total costs and total health outcomes of NOACs and VKAs comparisons in economic evaluation studies from the US, China, and the UK, according to different countries and cost years. Cost year refers to a specific base year in which economic evaluation studies measure and calculate the cost of drugs and medical services. All included studies used the CUA method, so QALY was used as the health outcome measure for comparison. The total costs and total health outcomes of the studies in different cost years in these different countries are shown in Figures 2–7, and the details are provided in Tables 2–4.

**TABLE 2:** Economic outcomes of included studies in the US.

References	Perspective	Patient	Cost year	Drugs	Total costs (USD/$)	QALYs	ICER	Cost-effective (Y/N)
Wu et al. (2021)	Payer	Elderly AF; Age: >75	2020	W	14,280	5.17		
				Edox	28,083	6.04	15,864	Y
				NOACs	29,515	5.53	42,318	Y
				Apix	30,649	5.48	52,800	N
				Dabi	32,177	5.42	71,587	N
				Riva	32,271	5.33	112,439	N
Wang et al. (2020)	Payer	AF; Age: ≥18; CHADS2 score: ≥2	2018	W	42,044	7.11		
				Riva	57,621	7.32	74,176	N
				Edox	51,188	7.36	51,188	N
				Apix	54,564	7.43	39,127	Y
				Dabi	53,962	7.45	35,055	Y
Salcedo et al. (2019)	Societal	Elderly AF; Age: 73; CHADS2 score: ≥2	2017	W	78,504	5.22		
				Riva	66,075	5.69	Dominant	Y
Hospodar et al. (2018)	Payer	AF with high risk of bleeding; Age: 65; CHADS2 score: ≥1	2015	W	199,393	7.9615		
				Edox	221,930	8.1382	127,544	N
				Apix	235,933	7.9451	Dominated	N
				D150	238,388	8.0165	709,000	N
				D110	242,878	8.0131	842,733	N
				Riva	237,483	7.3806	Dominated	N
Altawalbeh et al. (2018)	Healthcare system	AF with chronic kidney disease; Age: 40	2014	W	23,090	15.06		
				Apix	17,483	15.76	Dominant	Y
Vargas et al. (2018)	Societal	AF with AD; Age: 70; CHADS2 score: ≥2 in women; ≥1 in men	2015	W	268,367	4.58		
				Edox	274,970	4.656	86,882	Y
				Dabi	279,285	4.634	202,185	N
				Apix	280,981	4.651	177,662	N
				Riva	281,288	4.664	789,750	N
Hernandez et al. (2017)	Payer	AF at high risk of bleeding; Age: 65; CHADS2 score: ≥1	2012	W	184,252	7.9615		
				Edox	206,336	8.2462	77,569	Y
				Riva	212,579	8.1349	163,362	N
				Apix	214,614	8.3224	84,128	Y
				D150	220,927	8.304	107,080	Y
				D110	223,922	8.3058	115,219	Y
Shah et al. (2016)	Payer	AF; Age: 75; CHADS2 score: ≥1	2015	W	46,241	9.02		
				Apix	55,455	9.38	25,816	Y
				Edox	54,159	9.31	27,643	Y
				Dabi	56,425	9.35	31,435	Y
				Riva	58,889	9.24	574	Y
Nguyen et al. (2016)	Societal	AF at high risk for stroke; Age: 70; CHADS2 score: 3	2014	W	123,516	10.11		
				Edox	99,833	10.5	Dominant	Y
Salata et al. (2016)	Societal	AF at increased risk of stroke; Age: ≥65; CHADS2 score: ≥1	2013	W	95,528	8.998		
				D150	104,186	9.392	21,980	Y
Magnuson et al. (2015)	Healthcare system	AF; Age: 73; CHADS2 score: 2–6	2013	W	26,986	6.715		
				Edox	43,370	7.16	36,862	Y
Clemens et al. (2014)	Payer	AF	2013	W	21,366	7.75		
				Dabi	39,331	8.07	56,131	Y
You (2014)	Payer	AF; Age: 65; CHADS2 score: ≥2	2013	W	84,274	9.572		
				NOACs	98,524	9.97	35,804	Y
Harrington et al. (2013)	Societal	AF with high risk of stroke; Age: ≥70; CHADS2 score: ≥1	2012	W	77,813	7.97		
				Riva	78,738	8.26	3,190	Y
				D150	82,719	8.41	11,150	Y
				Apix	85,326	8.47	15,026	Y
Canestaro et al. (2013)	Societal	AF; Age: 70; CHADS2 score: 2	2011	W	49,638	5.87		
				Riva	84,192	6.18	111,465	N
				Apix	87,794	6.28	93,063	Y
				D110	88,994	6.15	140,557	N
Lee et al. (2012a)	Payer	AF; Age: 65; CHADS2 score: 3	2011	W	88,544	9.812		
				Riva	94,456	10.027	27,498	Y
Lee et al. (2012b)	Healthcare system	AF; Age: 65; CHADS2 score: 2.1	2011	W	90,225	10.89		
				Apix	87,592	11.23	Dominant	Y
Kamel et al. (2012a)	Societal	AF with secondary stroke risk; Age: 70	2010	W	378,500	3.91		
				Apix	381,700	4.19	11,400	Y
Shah and Gage (2011)	Payer	AF with stroke risk; Age: 70	2010	W	23,000	8.4		
				Dabi	43,700	8.65	86,000	N

USD, united states dollar; AF, atrial fibrillation; W, warfarin; Apix, apixaban; Riva, rivaroxaban; D110, dabigatran 110 mg; D150, dabigatran 150 mg; Dabi, dabigatran; Edox, edoxaban; Dominant, less costly and more effective; Dominated, more costly and less effective; Y, yes; N, no.

**TABLE 3:** Economic outcomes of included studies in China.

References	Perspective	Patient	Cost year	Drugs	Total costs (CNY/¥)	QALYs	ICER	Cost-effective (Y/N)
Zhou et al. (2022)	Healthcare system	AF	2020	W	63,168	8.7		
				Apix	60,487	9.23	Dominant	Y
				Riva	52,957	10.02	Dominant	Y
				D110	69,541	9.62	6,927	Y
				D150	108,924	10.53	25,004	Y
Yang (2021)	Healthcare system	AF; Age: 73; CHADS2 score: 3.47	2020	W	5,671	15.01		
				Riva	118,872	15.95	121,033	Y
Wei et al. (2021)	Payer	AF	2018	W	36,158	11.07		
				Riva	201,779	15.46	37,727	Y
				D110	160,585	12.40	93,555	Y
				D150	233,409	15.00	50,191	Y
Liu et al. (2021)	Healthcare system	AF; Age: 73; CHADS2 score: 3.5	2019	W	7,383	16.11		
				Riva	28,732	16.44	64,694	Y
Wang et al. (2022)	Payer	AF	2019	W	236,240	16.22		
				Riva	252,528	16.87	25,058	Y
Cui et al. (2020)	Payer	AF	2018	W	37,806	11.07		
				D110	167,906	12.4	97,820	Y
				D150	244,050	15	52,479	Y
				Riva	210,977	15.46	39,447	Y
Lv and Yang (2019)	Societal	AF; Age: 65; CHADS2 score: 3.2	2015	W	37,031	3.202		
				Apix	105,761	3.234	2,143,465	N
				Riva	105,645	3.226	2,882,448	N
				D150	105,055	3.232	2,271,356	N
Huang et al. (2019)	Healthcare system	AF; Age: 67; CHADS2 score: 3.1	2015	W	284,290	-		
				Riva	275,997	-	27,952	Y
Wu et al. (2016a)	Societal	AF with a high risk of stroke; CHADS2 score: ≥2	2014	W	46,587	6.152		
				Apix	309,735	7.01	306,699	N
				D110	163,586	6.812	177,271	N
				D150	199,876	6.932	196,524	N
				Riva	582,710	6.877	739,480	N
Wu et al. (2016b)	Societal	AF with high risk of stroke; Age: ≥18; CHADS2 score: ≥2	2014	W	40,126	5.614		
				Apix	271,826	6.256	360,903	N
Chen and Han (2016)	Societal	NVAF with stroke	2014	W	30,904	12.88		
				D150	168,085	13.26	361,003	N
				Riva	382,419	13.03	2,343,433	N
Wan et al. (2014)	Payer	AF; Age: 69.32; CHADS2 score: 2.0	2013	W	35,732	6. 98		
				Dabi	109,305	7. 18	375,291	N
Wu et al. (2014)	Healthcare system	AF; Age: ≥75; CHADS2 score: 3	2012	W	51,085	9.24		
				Riva	673,952	9.69	1,384,147	N

CNY, chinese yuan; AF, atrial fibrillation; W, warfarin; Apix, apixaban; Riva, rivaroxaban; D110, dabigatran 110 mg; D150, dabigatran 150 mg; Dabi, dabigatran; Edox, edoxaban; Dominant, less costly and more effective; Y, yes; N, no.

**TABLE 4:** Economic outcomes of included studies in the UK.

References	Perspective	Patient	Cost year	Drugs	Total costs (GBP/£)	QALYs	ICER	Cost-effective (Y/N)
Bowrin et al. (2020a)	Healthcare system	AF; Age: 75; CHADS2 score: 10% (=1) and 90% (≥2)	2018	VKAs	9,889	5.96		
				Riva	11,598	6.08	14,437	Y
				Apix	11,734	6.06	20,101	N
Thom et al. (2019)	Healthcare system	AF; Age: 70	2017	W	24,418	5.166		
				Apix	23,340	5.488	Dominant	Y
				Dabi	23,064	5.416	Dominant	Y
				Edox	23,985	5.405	Dominant	Y
				Riva	24,841	5.451	1,484	Y
Zheng et al. (2014)	Payer	AF; Age: 71; CHADS2 score: 2.1	2013	W	24,680	7.36		
				Dabi	23,342	7.68	Dominant	Y
				Apix	24,014	7.63	Dominant	Y
				Riva	25,220	7.47	4,909	Y
Dorian et al. (2014)	Healthcare system	AF; Age: 70	2011	W	6,920	6.08		
				Apix	9,078	6.26	11,909	Y
Pink et al. (2014)	Healthcare system	AF; Age: 72.3; CHADS2 score: 2.1	2011	W	5,880	5.721		
				Riva	9,112	5.817	33,666	N
				Dabi	8,426	5.827	24,018	Y
				Apix	8,437	5.851	19,669	Y
Kansal et al. (2012)	Healthcare system	AF; Age: 69; CHADS2 score: 2.1	2010	W	18,474	7.82		
				Dabi	19,645	8.06	4,831	Y
Kamel et al. (2012b)	Societal	AF with stroke risk; Age: ≥70	2010	W	NA	3.91		
				Dabi	NA	4.27	25,000	Y

GBP, great british pound; AF, atrial fibrillation; W, warfarin; Apix, apixaban; Riva, rivaroxaban; D110, dabigatran 110 mg; D150, dabigatran 150 mg; Dabi, dabigatran; Edox, edoxaban; Dominant, less costly and more effective; Dominated, more costly and less effective; Y, yes; N, no.

Although there were significant differences in total costs between different countries due to differences in economic development level and healthcare resources, making direct comparison not feasible, some trends are worth exploring. Overall, the total cost measured varies widely between countries and different years, but in the majority of studies, the total costs of the NOACs regimen were higher than the total costs of the VKAs regimen. Moreover, the total costs of studies conducted in the UK showed a relatively concentrated distribution, mainly ranging from €5,000 to €25,000. Kamel et al.'s study (Kamel et al., 2012a) in the US, conducted from the societal perspective, targeted AF patients with a history of stroke or TIA and measured a high total cost of approximately $380,000. Vargas et al.'s study (Vargas et al., 2018), also from the societal perspective, measured a total cost of about $280,000 AF patients with Alzheimer’s disease. And the total cost measured by two studies with a target population of 65-year-old AF patients with high bleeding risk was also relatively high (around $200,000) (Hospodar et al., 2018; Hernandez et al., 2017), compared to other studies (mainly setting target population age above 70 years). The remaining US studies measured more concentrated costs, ranging from $20,000 to $100,000.

In studies carried out in China, Wu et al. (2014), Chen and Han (2016), and Wu et al. (2016a) measured the overall cost of NOACs as significantly higher than other studies, ¥670,000, ¥380,000, and ¥580,000, respectively. These three studies were early studies of rivaroxaban, as rivaroxaban had just entered the Chinese market, NOACs were not yet fully competitive, and the high drug price of rivaroxaban itself led to a very high total cost of rivaroxaban regimens. Among them, Wu et al. (2016a) was also a study from a social perspective, including direct medical costs (DMCs) and direct non-medical costs (DNMCs). The total cost measured by the remaining studies was distributed within ¥300,000.

In addition, we also found that in the UK and the US, the total cost of NOACs and VKAs was relatively close, while in China the two were quite different, especially in earlier studies, the total cost of NOACs was significantly higher than the total cost of VKAs (Wu et al., 2016a; Chen and Han, 2016; Wu et al., 2014), mainly due to the high prices of NOACs when they were first introduced to the Chinese market, while VKAs such as warfarin had been used in China for many years and had a lower price. The cost of drugs, especially the cost of NOACs is the main component of the total cost, and the change in its price directly affects the change of the total cost. Observing the change in the total cost of Chinese studies over time, it can be found that there is a clear downward trend in the total cost of the NOACs group, which might be attributed to drug price adjustments by pharmaceutical companies and the implementation of volume-based procurement policy in China starting from 2018, which includes apixaban, rivaroxaban, and dabigatran.

Regarding total health outcomes, different studies showed some differences, which might be related to baseline characteristics of the target population such as age, stroke risk, bleeding risk, and comorbidities. In general, most studies showed that the total health outcomes of NOACs were higher than VKAs, reflecting the better efficacy and safety of NOACs. In the US, one study (Altawalbeh et al., 2018) had a significantly higher total health outcome (16 QALYs) due to the lower average age of the target population (around 40 years) compared to other studies (mainly setting target population age above 70 years). The total health outcomes of other studies in the US were more concentrated, mainly ranging from 4 QALYs to 11 QALYs. In the UK, the total health outcomes of different studies were relatively concentrated, mainly ranging from 4 QALYs to 8 QALYs. In China, the total health outcomes were more dispersed among different studies, with a wide range of 3 QALYs to 16 QALYs. In recent years, the total health outcomes measured in China were generally higher than in earlier years, which might be related to variations in patient baseline characteristics, clinical efficacy data sources, and study quality.

### 3.5 Results of economic evaluations

The systematic review included a total of 218 comparisons between NOACs and VKAs in all 103 included studies. Among them, 157 studies (72.0%) showed that NOACs were cost-effective compared with VKAs, while the remaining 61 studies (28.0%) favored VKAs. In HICs, 84.2% (139/165) of the studies showed that NOACs were cost-effective compared with VKAs, while in LMICs, 66.0% (35/53) favored VKAs. This suggests that the economic development level of the country may influence cost-effectiveness results.

A multivariate logistic regression equation was constructed to explore the factors influencing the cost-effectiveness results of NOACs and VKAs across all included studies, considering variables such as the country’s economic development level, study perspective, and funding status. Collinearity diagnostics confirmed that there were no significant multicollinearity issues among the independent variables. The results showed that compared to LMICs, NOACs were more likely to be cost-effective in HICs, and this difference was statistically significant (OR = 6.080, 95% CI 2.474–14.942, P < 0.001). Studies conducted from the perspective of payers and healthcare systems were more likely to show that NOACs were cost-effective compared to studies from the societal perspective, and this difference was statistically significant (OR = 8.425, 95% CI 3.347–21.211, P < 0.001). Moreover, the cycle length was also a factor influencing the cost-effectiveness, with studies setting a cycle length of 3 months or less more likely to show that NOACs were cost-effective compared to studies with a one-year cycle length, and this difference was statistically significant (OR = 2.571, 95% CI 1.071–6.171, P = 0.035). The results of the regression analysis are shown in Table 5.

**TABLE 5:** The results of the regression analysis.

Item (N)	B	SE	P - value	OR	OR - 95% CI
Economic development level					
LMICs (53)^a^					
HICs (165)	1.805	0.459	<0.001	6.080	[2.474, 14.942]
Perspective					
Societal (51)^a^					
Payer/Healthcare system (167)	2.131	0.471	<0.001	8.425	[3.347, 21.211]
Half-cycle correction					
N (194)^a^					
Y (24)	−1.025	0.573	0.074	0.359	[0.117, 1.104]
Cycle length					
1 year (54)^a^					
≤3 months (164)	0.944	0.447	0.035	2.571	[1.071, 6.171]
Funding					
Industry (73)^a^					
Government/Scientific research institute/no funding (145)	−0.203	0.461	0.660	0.816	[0.331, 2.016]
Literature quality (compliance rate)					
≥80% (89)^a^					
<80% (129)	−0.170	0.470	0.718	0.844	[0.336, 2.119]
Cost Year	0.064	0.071	0.367	1.066	[0.927, 1.227]

^a^ Reference; the dependent variable: 1: NOACs are cost-effective, VKAs are not cost-effective; 0: NOACs are not cost-effective, VKAs are cost-effective. Cost year: a specific base year in which economic evaluation studies measure and calculate the cost of drugs and medical services.

## 4 Discussion

In this study, we conducted a detailed discussion on the cost, health outcomes, and cost-effectiveness results of existing pharmacoeconomic evaluations comparing NOACs to VKAs in AF patients. The results revealed significant variations in total costs measured across different countries and years, yet the total costs of NOACs were generally higher than that of VKAs. The overall health outcomes of treatment strategies were influenced by the patients’ baseline characteristics, but the total health output of NOACs tended to be higher than that of VKAs. Overall, NOACs regimens generally had higher total costs but also yielded greater overall health outcomes compared to VKAs regimens. In terms of cost-effectiveness, on the whole, NOACs were found to be more cost-effective compared to VKAs. However, this conclusion was influenced by factors such as the country’s economic development level, study perspective, and model cycle length.

Examining the total costs in the US, China, and the UK, we observed differences in cost outcomes for the same country in different years. These differences might be attributed to factors such as the study perspective, patients’ baseline characteristics, and drug prices. Different study perspectives include different cost measurement scopes. Studies from the societal perspective tended to have higher total costs since they considered not only DMCs but also DNMCs and ICs. For instance, studies conducted by Kamel et al. (2012a), Vargas et al. (2018), and Wu et al. (2016a) from the societal perspective included DNMCs and ICs, resulting in higher total costs compared to other studies in the same country.

Differences in patients’ baseline characteristics in different studies also contributed to variations in total costs and total health outcomes within the same country (Altawalbeh et al., 2018; Vargas et al., 2018; Hernandez et al., 2017; Kamel et al., 2012a). This review included studies that mainly focused on elderly AF patients with certain stroke risks, and some studies also considered bleeding risk, history of stroke, and comorbidities, all of which could cause costs, health outcomes, and cost-effectiveness results differences. Some studies conducted subgroup analyses (Wu et al., 2021; Wang et al., 2020; Shah et al., 2016; Magnuson et al., 2015; Clemens et al., 2014; Liu et al., 2021; Dorian et al., 2014; Lekuona et al., 2019; van Hulst et al., 2018; Vilain et al., 2017; Zhao et al., 2016; Krejczy et al., 2015; Rognoni et al., 2014; Jarungsuccess and Taerakun, 2014; Krejczy et al., 2014; Davidson et al., 2013; Freeman et al., 2011), and the results showed that in different subgroups classified based on age, stroke risk, and bleeding risk, the safety, effectiveness, and economic benefits varied, indicating that cost-effectiveness may heavily depend on patients' characteristics. This suggests the importance and necessity of conducting relevant pharmacoeconomic evaluation studies based on specific patient populations and research contexts, as well as being cautious about the applicability and generalizability of cost-effectiveness conclusions.

The drug price, especially for innovative drugs like NOACs, is a major component of the total treatment cost, and it has a crucial impact on cost-effectiveness results. Through Chinese studies in different years, it was observed that when NOACs were first launched in China, the price of NOACs was initially high due to insufficient market competition, while VKAs such as warfarin have been used for many years, and the price has been stable and cheap, resulting in the total cost of NOACs measured in early studies was significantly higher than the total cost measured by VKAs (Wu et al., 2016a; Chen and Han, 2016; Wu et al., 2014). However, with the gradual filling of market competition, the initiative of pharmaceutical companies to reduce prices, and most importantly, the implementation of centralized procurement in China, the drug prices of NOACs in China have dropped significantly. The changes in drug prices have influenced the variations in total costs, thereby exerting a significant impact on cost-effectiveness. In our findings, during the initial years of NOACs entering the Chinese market, several studies in China consistently indicated that NOACs were not cost-effective compared to VKAs (Wu et al., 2016a; Wu et al., 2016b; Chen and Han, 2016; Wan et al., 2014; Wu et al., 2014). However, in recent years, especially after 2018, multiple studies (Zhou et al., 2022; Yang, 2021; Wei et al., 2021; Liu et al., 2021) have conducted analyses using the adjusted drug prices and consistently demonstrated that NOACs are cost-effective. China bears the heaviest burden of AF globally, with the incidence rate, mortality rate, and disability-adjusted life years ranking first in the world, accounting for approximately one-fourth of the global disease burden (Roth et al., 2020). In comparison to VKAs, NOACs have shown clear cost-effectiveness and have been widely used in China, relieving the burden on patients and the healthcare system. Nevertheless, the comparison of cost-effectiveness among different NOACs has become a focus in the Chinese healthcare system. However, there is a severe lack of research on this topic, and it is imperative to conduct further studies in the future.

Through multivariate regression analysis, we found that the country’s economic development level, study perspective, and model cycle length significantly influenced the cost-effectiveness results of NOACs and VKAs in AF patients. A previous meta-analysis study (Noviyani et al., 2022) that classified existing cost-effectiveness results of NOACs compared to VKAs based on different factors also found that the country’s socioeconomic status and study perspective might affect the cost-effectiveness of NOACs compared to VKAs, with conclusions similar to this study. Both meta-analysis and regression analysis, two different methods, lead to similar results, which to some extent validate the reliability and robustness of this study’s conclusions.

The country’s economic development level had a significant impact on cost-effectiveness results. For HICs, the probability of NOACs being cost-effective was higher, while for LMICs, NOACs may not be cost-effective, and VKAs were more likely to be cost-effective. This may be attributed to different preferences for treatment effectiveness, QALYs, and resource allocation in different countries and regions. HICs may be more inclined to consider higher treatment efficacy and life value in their decision-making, thus setting a more lenient threshold. Conversely, LMICs, with limited healthcare resources, may prioritize economic efficiency, leading to lower cost-effectiveness thresholds. Compared with traditional VKAs such as warfarin, NOACs are relatively more expensive, and patients with AF usually require lifelong anticoagulant therapy, resulting in significantly higher total costs for NOACs regimens and a greater economic burden on patients and healthcare systems. Although NOACs provide more health benefits to patients compared to VKAs, the cost-effectiveness thresholds set by LMICs are generally lower, potentially making NOACs less cost-effective (Aghoram et al., 2022; Dilokthornsakul et al., 2020; Rattanachotphanit et al., 2019).

The regression analysis also found that different study perspectives significantly influenced cost-effectiveness results. Studies conducted from a societal perspective generally suggested lower probabilities of NOACs being cost-effective compared to VKAs, while studies conducted from a healthcare system perspective or payer perspective tended to indicate higher probabilities of NOACs being cost-effective. Although the multivariate regression model partially controlled for variables such as the country’s economic level, the societal perspective studies were less with the majority from LMICs and little from HICs, which might have impacted the results. Additionally, the societal perspective includes not only DMCs but also DNMCs and indirect costs (ICs). The case of warfarin requires regular monitoring and dose adjustments based on blood clotting indicators like INR, but this monitoring process is relatively mature and cheap. Furthermore, AF patients are often elderly, leading to lower ICs related to workforce losses. The DNMCs such as transportation expenses caused by INR tests may not substantially increase due to the regular follow-ups required by the patients themselves. In the comparison between the two treatment strategies, the drug cost of NOACs remains a major component, and their relatively higher prices compared to VKAs, though along with the convenience of not requiring monitoring, do not necessarily lead to significant cost savings from a societal perspective.

Furthermore, the cycle length used in the analysis also significantly impacted cost-effectiveness results. Studies with a cycle length of 3 months or less were associated with a higher probability of NOACs being cost-effective compared to a one-year cycle. This could be attributed to the fact that AF patients receiving anticoagulant therapy require follow-ups every 1–3 months, and patients using warfarin typically undergo INR testing every 4–12 weeks. As such, setting the cycle length to 1–3 months aligns better with clinical needs and allows for more accurate modeling of event occurrence rates and resource utilization, resulting in better capture of healthcare resource consumption and health outcomes.

Regarding industry funding, this study’s multivariate regression model did not find significant impacts on cost-effectiveness results. However, a previous study (Xie and Zhou, 2022) found that industry funding significantly influenced cost-effectiveness results in the cardiovascular disease field, and led to sponsorship and publication bias, suggesting that industry-funded studies are more likely to find the intervention group as cost-effective compared to non-industry-funded studies. In the included studies, 82.2% (60/73) of the studies with industry sponsorship showed NOACs to be more cost-effective, while this proportion decreased to 66.9% (97/145) in studies without industry sponsorship, suggesting a trend of sponsorship bias. Although industry sponsorship had a significant impact on cost-effectiveness results in the chi-square test and univariate logistic regression analysis, it became non-significant after adding other covariates, possibly due to the small sample size and other confounding factors. Nevertheless, it is essential to maintain vigilance against sponsorship and publication bias, given the widespread acceptance and utilization of cost-effectiveness evidence in price negotiations and the formulation of healthcare insurance coverage policies. To ensure the reliability of cost-effectiveness evidence, it is necessary and important to conduct CEA through independent organizations.

Currently, the clinical efficacy and safety of NOACs relative to VKAs have been established, and many countries’ guidelines prioritize NOACs as the preferred anticoagulants for stroke prevention in atrial fibrillation patients (Hindricks et al., 2021; Steffel et al., 2021). However, the high cost of NOACs limits their clinical application to some extent. At present prices, NOACs are cost-effective only in HICs, while they may not be cost-effective in LMICs (Aghoram et al., 2022; Dilokthornsakul et al., 2020; Rattanachotphanit et al., 2019). In China, the implementation of centralized procurement policies has reduced drug prices, leading to an improvement in the cost-effectiveness of NOACs and increased accessibility for patients. In contrast, in other LMICs, the cost of NOACs remains significantly higher than that of warfarin, limiting their clinical uptake. Due to limited healthcare resources and lower economic development levels, disease management for AF is inadequate in these countries (Aghoram et al., 2022; Dilokthornsakul et al., 2020; Rattanachotphanit et al., 2019). Moreover, the limited availability of cost-effectiveness data in LMICs further complicates informed decision-making in these settings.

However, with the expiration of market monopolies (dabigatran, apixaban, edoxaban, and rivaroxaban in 2018, 2020, 2022, and 2023, respectively) and the gradual introduction of generic drugs, market competition is expected to become more robust. This is anticipated to lead to a decrease in the prices of NOACs and subsequently improve their cost-effectiveness. As a result, patients in LMICs will gain increased accessibility to NOACs, significantly alleviating the burden of disease and enhancing patients’ health outcomes. Policymakers and pharmaceutical companies should collaborate, taking into account the impact of economic development on cost-effectiveness, and adopt proactive pricing strategies and cooperative models including drug price negotiations, generic drug introduction, and the implementation of centralized procurement to ensure the affordability and accessibility of medications. Clinicians and payers should be aware of country-specific cost-effectiveness evidence to guide optimal treatment choices, balancing clinical benefits with economic sustainability.

Future research should focus on generating real-world economic evaluations of NOACs in LMICs, incorporating local cost structures, healthcare utilization patterns, and patient adherence data. Additionally, studies assessing the long-term budget impact of NOACs adoption under different financing models could provide valuable insights for policymakers. Addressing these research gaps will be critical to ensuring that cost-effectiveness assessments reflect the realities of diverse healthcare systems, ultimately supporting more equitable access to optimal anticoagulation therapy worldwide.

Limitations: First, this review performed logistic regression analysis on the included studies' cost-effectiveness results, despite controlling for covariates such as the country’s economic development level, study perspective, and study quality, there were still many factors contributing to heterogeneity, resulting from differences in patients’ baseline characteristics, healthcare systems, etc. As such, the study can only observe general trends in cost-effectiveness factors, and specific situations may vary due to research backgrounds, patients’ baseline characteristics, and other factors. Furthermore, the choice of cost-effectiveness threshold can significantly impact cost-effectiveness results. Different countries have different thresholds, with the US usually setting thresholds between $50,000 to $150,000, the UK setting thresholds between £10,000 to £30,000, and others usually using 1–3 times the GDP *per capita* as the threshold. However, some study results fell within the set threshold range above. This review used the threshold set by the included study itself as a reference to judge whether it is cost-effective or not. The number of these studies is small, and their impact on the overall trend is expected to be random, not significantly affecting the general trends observed in this review.

## 5 Conclusion

Compared to VKAs strategies, NOACs strategies lead to greater health benefits and higher costs. In HICs, NOACs are generally cost-effective, while in LMICs, VKAs may still be an attractive strategy. Drug prices, particularly for innovative drugs, are a critical factor in total treatment costs and can significantly impact cost-effectiveness results. China’s centralized drug procurement policy has lowered NOACs prices and improved their cost-effectiveness. Moreover, the study perspective, patients’ baseline characteristics, and model settings influence the cost, health outcomes, and cost-effectiveness results. Conducting relevant pharmacoeconomic evaluations based on specific populations and study contexts is essential. It is crucial to be mindful of the applicability and generalizability of cost-effectiveness conclusions.

## Data Availability

The original contributions presented in the study are included in the article/Supplementary Material, further inquiries can be directed to the corresponding authors.

## References

[B1] Ademi Z. Pasupathi K. Liew D. (2015). Cost-effectiveness of apixaban compared to warfarin in the management of atrial fibrillation in Australia. Eur. J. Prev. Cardiol. 22 (3), 344–353. 10.1177/2047487313514019 24281250

[B2] Agbor V. N. Aminde L. N. Tianyi F. L. Mbanga C. M. Petnga S. N. Ditah C. (2019). Atrial fibrillation among adults with heart failure in sub-Saharan Africa-prevalence, incidence and all-cause mortality: a systematic review and meta-analysis protocol. BMJ Open 9 (2), e022320. 10.1136/bmjopen-2018-022320 PMC6410087 30808667

[B3] Aghoram R. Kumar S. M. Rajasulochana S. R. Kar S. S. Aggarwal R. (2022). Cost-utility analysis of dabigatran and warfarin for stroke prevention among patients with nonvalvular atrial fibrillation in India. Value Health Reg. Issues 31, 119–126. 10.1016/j.vhri.2022.04.007 35667196

[B4] Altawalbeh S. M. Alshogran O. Y. Smith K. J. (2018). Cost-utility analysis of apixaban versus warfarin in atrial fibrillation patients with chronic kidney disease. Value Health 21 (12), 1365–1372. 10.1016/j.jval.2018.06.009 30502779

[B5] Andrikopoulos G. K. Fragoulakis V. Maniadakis N. (2013). Economic evaluation of dabigatran etexilate in the management of atrial fibrillation in Greece. Hell. J. Cardiol. 54 (4), 289–300. 23912921

[B6] Athanasakis K. Boubouchairopoulou N. Karampli E. Tarantilis F. Savvari P. Bilitou A. (2017). Cost effectiveness of apixaban versus warfarin or aspirin for stroke prevention in patients with atrial fibrillation: a Greek perspective. Am. J. Cardiovasc Drug 17 (2), 123–133. 10.1007/s40256-016-0204-1 27882517

[B7] Barón E. G. Escolar Albaladejo G. Zamorano J. L. Betegón Nicolás L. Canal Fontcuberta C. de Salas-Cansado M. (2015). Cost-effectiveness analysis comparing apixaban and acenocoumarol in the prevention of stroke in patients with nonvalvular atrial fibrillation in Spain. Rev. Española Cardiol. English Ed. 68 (8), 680–690. 10.1016/j.rec.2014.08.010 25498373

[B8] Bowrin K. Briere J. Fauchier L. Coleman C. Millier A. Toumi M. (2020b). Real-world cost-effectiveness of rivaroxaban compared with vitamin K antagonists in the context of stroke prevention in atrial fibrillation in France. Plos One 15 (1), e0225301. 10.1371/journal.pone.0225301 31978044 PMC6980557

[B9] Bowrin K. Briere J. B. Levy P. Millier A. Tardu J. Toumi M. (2020a). Real-world cost-effectiveness of rivaroxaban and apixaban vs VKA in stroke prevention in non-valvular atrial fibrillation in the UK. J. Mark. Access Health Policy 8 (1), 1782164. 10.1080/20016689.2020.1782164 32944199 PMC7482848

[B10] Camm A. J. Kirchhof P. Lip G. Y. Schotten U. Savelieva I. Ernst S. (2010). Guidelines for the management of atrial fibrillation: the task force for the management of atrial fibrillation of the European society of cardiology (ESC). Eur. Heart J. 31 (19), 2369–2429. 10.1093/eurheartj/ehq278 20802247

[B11] Canestaro W. J. Patrick A. R. Avorn J. Ito K. Matlin O. S. Brennan T. A. (2013). Cost-effectiveness of oral anticoagulants for treatment of atrial fibrillation. Circ. Cardiovasc Qual. Outcomes 6 (6), 724–731. 10.1161/CIRCOUTCOMES.113.000661 24221832

[B12] Carles M. Brosa M. Souto J. C. Garcia-Alamino J. M. Guyatt G. Alonso-Coello P. (2015). Cost-effectiveness analysis of dabigatran and anticoagulation monitoring strategies of vitamin K antagonist. Bmc Health Serv. Res. 15 (1), 289. 10.1186/s12913-015-0934-9 26215871 PMC4515878

[B13] Chang C. H. Yang Y. H. Chen J. H. Lin L. J. (2014). Cost-effectiveness of dabigatran etexilate for the prevention of stroke and systemic embolism in atrial fibrillation in Taiwan. Thromb. Res. 133 (5), 782–789. 10.1016/j.thromres.2014.02.024 24642004

[B14] Chen Y. F. Han H. N. (2016). Economic evaluation of dabigatran, rivaroxaban and warfarin in preventing stroke in patients with atrial fibrillation. Chin. J. New Drugs 25 (11), 1216–1224.

[B15] Chevalier J. Delaitre O. Hammes F. de Pouvourville G. (2014). Cost-effectiveness of dabigatran versus vitamin K antagonists for the prevention of stroke in patients with atrial fibrillation: a French payer perspective. Arch. Cardiovasc Dis. 107 (6-7), 381–390. 10.1016/j.acvd.2014.04.009 24973113

[B16] Choi J. H. Kim W. Kim Y. T. Cho J. Shin S. Y. Kim C. (2022). Cost-effectiveness of direct oral anticoagulant vs. warfarin among atrial fibrillation patients with intermediate stroke risk. Front. Cardiovasc Med. 9, 849474. 10.3389/fcvm.2022.849474 35479283 PMC9035745

[B17] Clemens A. Peng S. Brand S. Brueckmann M. Kansal A. Lim J. (2014). Efficacy and cost-effectiveness of dabigatran etexilate versus warfarin in atrial fibrillation in different age subgroups. Am. J. Cardiol. 114 (6), 849–855. 10.1016/j.amjcard.2014.06.015 25103918

[B18] Connolly S. J. Ezekowitz M. D. Yusuf S. Eikelboom J. Oldgren J. Parekh A. (2009). Dabigatran versus warfarin in patients with atrial fibrillation. New Engl. J. Med. 361 (12), 1139–1151. 10.1056/NEJMoa0905561 19717844

[B19] Coyle D. Coyle K. Cameron C. Lee K. Kelly S. Steiner S. (2013). Cost-effectiveness of new oral anticoagulants compared with warfarin in preventing stroke and other cardiovascular events in patients with atrial fibrillation. Value Health 16 (4), 498–506. 10.1016/j.jval.2013.01.009 23796283

[B20] Cui C. Liu Y. Cui X. L. Wei H. T. (2020). Oral anticoagulants for stroke prevention in atrial fibrillation:a cost-effectiveness analysis under different medical insurances in China. Clin. Medicat. J. 18 (12), 63–68. 10.3969/j.issn.1672-3384.2020.12.013

[B21] Davidson T. Husberg M. Janzon M. Oldgren J. Levin L. (2013). Cost-effectiveness of dabigatran compared with warfarin for patients with atrial fibrillation in Sweden. Eur. Heart J. 34 (3), 177–183. 10.1093/eurheartj/ehs157 22733833

[B22] Degeling K. Vu M. Koffijberg H. Wong H. Koopman M. Gibbs P. (2020). Health economic models for metastatic colorectal cancer: a methodological review. Pharmacoeconomics 38 (7), 683–713. 10.1007/s40273-020-00908-4 32319026

[B23] de Jong L. A. Groeneveld J. Stevanovic J. Rila H. Tieleman R. G. Huisman M. V. (2019). Cost-effectiveness of apixaban compared to other anticoagulants in patients with atrial fibrillation in the real-world and trial settings. Plos One 14 (9), e0222658. 10.1371/journal.pone.0222658 31527894 PMC6748426

[B24] de Pouvourville G. Blin P. Karam P. (2020). The contribution of real-world evidence to cost-effectiveness analysis: case study of Dabigatran etexilate in France. Eur. J. Health Econ. 21 (2), 235–249. 10.1007/s10198-019-01123-5 31650440

[B25] Dilokthornsakul P. Nathisuwan S. Krittayaphong R. Chutinet A. Permsuwan U. (2020). Cost-effectiveness analysis of non-vitamin K antagonist oral anticoagulants versus warfarin in Thai patients with non-valvular atrial fibrillation. Heart Lung Circ. 29 (3), 390–400. 10.1016/j.hlc.2019.02.187 31000364

[B26] Dorian P. Kongnakorn T. Phatak H. Rublee D. A. Kuznik A. Lanitis T. (2014). Cost-effectiveness of apixaban vs. current standard of care for stroke prevention in patients with atrial fibrillation. Eur. Heart J. 35 (28), 1897–1906. 10.1093/eurheartj/ehu006 24513791 PMC4104492

[B27] Dwiprahasto I. Kristin E. Endarti D. Pinzon R. T. Yasmina A. Thobari J. A. (2019). Cost effectiveness analysis of rivaroxaban compared to warfarin and aspirin for stroke prevention atrial fibrillation (SPAF) in the Indonesian healthcare setting. Indones. J. Pharm. 30 (1), 74. 10.14499/indonesianjpharm30iss1pp74

[B28] Escobar Cervantes C. Martí-Almor J. Cabeza A. I. P. Bowrin K. Llorac Moix A. Genís Gironès M. (2022). Real-world cost-effectiveness analysis of NOACs versus VKA for stroke prevention in Spain. Plos One 17 (4), e0266658. 10.1371/journal.pone.0266658 35443000 PMC9020681

[B29] Freeman J. V. Zhu R. P. Owens D. K. Garber A. M. Hutton D. W. Go A. S. (2011). Cost-effectiveness of dabigatran compared with warfarin for stroke prevention in atrial fibrillation. Ann. Intern Med. 154 (1), 1–11. 10.7326/0003-4819-154-1-201101040-00289 21041570

[B30] Giorgi M. A. Caroli C. Giglio N. D. Micone P. Aiello E. Vulcano C. (2015). Estimation of the cost-effectiveness of apixaban versus vitamin K antagonists in the management of atrial fibrillation in Argentina. Health Econ. Rev. 5 (1), 52. 10.1186/s13561-015-0052-8 26112219 PMC4480270

[B31] Giugliano R. P. Ruff C. T. Braunwald E. Murphy S. A. Wiviott S. D. Halperin J. L. (2013). Edoxaban versus warfarin in patients with atrial fibrillation. New Engl. J. Med. 369 (22), 2093–2104. 10.1056/NEJMoa1310907 24251359

[B32] Granger C. B. Alexander J. H. McMurray J. J. V. Lopes R. D. Hylek E. M. Hanna M. (2011). Apixaban versus warfarin in patients with atrial fibrillation. New Engl. J. Med. 365 (11), 981–992. 10.1056/NEJMoa1107039 21870978

[B33] Hallinen T. Soini E. Asseburg C. Linna M. Eloranta P. Sintonen S. (2021). Cost-effectiveness of apixaban versus other direct oral anticoagulants and warfarin in the prevention of thromboembolic complications among Finnish patients with non-valvular atrial fibrillation. Clin. Outcomes Res. 13, 745–755. 10.2147/CEOR.S317078 PMC8370583 34413661

[B34] Hallinen T. Soini E. J. Linna M. Saarni S. I. (2016). Cost-effectiveness of apixaban and warfarin in the prevention of thromboembolic complications among atrial fibrillation patients. Springerplus 5 (1), 1354. 10.1186/s40064-016-3024-5 27588247 PMC4988956

[B35] Harrington A. R. Armstrong E. P. Nolan P. J. Malone D. C. (2013). Cost-effectiveness of apixaban, dabigatran, rivaroxaban, and warfarin for stroke prevention in atrial fibrillation. Stroke 44 (6), 1676–1681. 10.1161/STROKEAHA.111.000402 23549134

[B36] Hernandez I. Smith K. J. Zhang Y. (2017). Cost-effectiveness of non-vitamin K antagonist oral anticoagulants for stroke prevention in patients with atrial fibrillation at high risk of bleeding and normal kidney function. Thromb. Res. 150, 123–130. 10.1016/j.thromres.2016.10.006 27771008

[B37] Hersi A. S. Osenenko K. M. Kherraf S. A. Aziz A. A. Sambrook R. J. (2019). Cost-effectiveness of apixaban for stroke prevention in non-valvular atrial fibrillation in Saudi Arabia. Ann. Saudi Med. 39 (4), 265–278. 10.5144/0256-4947.2019.265 31381381 PMC6838647

[B38] Hindricks G. Potpara T. Dagres N. Arbelo E. Bax J. J. Blomström-Lundqvist C. (2021). 2020 ESC Guidelines for the diagnosis and management of atrial fibrillation developed in collaboration with the European Association for Cardio-Thoracic Surgery (EACTS): the Task Force for the diagnosis and management of atrial fibrillation of the European Society of Cardiology (ESC) Developed with the special contribution of the European Heart Rhythm Association (EHRA) of the ESC. Eur. Heart J. 42 (5), 373–498. 10.1093/eurheartj/ehaa612 32860505

[B39] Hori M. Tanahashi N. Akiyama S. Kiyabu G. Dorey J. Goto R. (2020). Cost-effectiveness of rivaroxaban versus warfarin for stroke prevention in non-valvular atrial fibrillation in the Japanese healthcare setting. J. Med. Econ. 23 (3), 252–261. 10.1080/13696998.2019.1688821 31687870

[B40] Hospodar A. R. Smith K. J. Zhang Y. Hernandez I. (2018). Comparing the cost effectiveness of non-vitamin K antagonist oral anticoagulants with well-managed warfarin for stroke prevention in atrial fibrillation patients at high risk of bleeding. Am. J. Cardiovasc Drug 18 (4), 317–325. 10.1007/s40256-018-0279-y 29740750

[B41] Huang B. Y. Zeng W. Q. Tan G. L. Liu X. Q. Lun Y. N. Chen W. Y. (2019). Cost-effectiveness analysis of rivaroxaban versus warfarin for stroke prevention in patients with nonvalvular atrial fibrillation. Chin J Clin. Ration. Drug Use 12 (16), 57–58. 10.15887/j.cnki.13-1389/r.2019.16.029

[B42] Husereau D. Drummond M. Augustovski F. de Bekker-Grob E. Briggs A. H. Carswell C. (2022). Consolidated health economic evaluation reporting standards 2022 (CHEERS 2022) statement: updated reporting guidance for health economic evaluations. Value Health 25 (1), 3–9. 10.1016/j.jval.2021.11.1351 35031096

[B43] Janzic A. Kos M. (2015). Cost effectiveness of novel oral anticoagulants for stroke prevention in atrial fibrillation depending on the quality of warfarin anticoagulation control. Pharmacoeconomics 33 (4), 395–408. 10.1007/s40273-014-0246-7 25512096

[B44] Jarungsuccess S. Taerakun S. (2014). Cost-utility analysis of oral anticoagulants for nonvalvular atrial fibrillation patients at the police general hospital, Bangkok, Thailand. Clin. Ther. 36 (10), 1389–1394. 10.1016/j.clinthera.2014.08.016 25267360

[B45] Kamae I. Hashimoto Y. Koretsune Y. Tanahashi N. Murata T. Phatak H. (2015). Cost-effectiveness analysis of apixaban against warfarin for stroke prevention in patients with nonvalvular atrial fibrillation in Japan. Clin. Ther. 37 (12), 2837–2851. 10.1016/j.clinthera.2015.10.007 26608819

[B46] Kamel H. Easton J. D. Johnston S. C. Kim A. S. (2012a). Cost-effectiveness of apixaban vs warfarin for secondary stroke prevention in atrial fibrillation. Neurology 79 (14), 1428–1434. 10.1212/WNL.0b013e31826d5fe8 22993279 PMC3525294

[B47] Kamel H. Johnston S. C. Easton J. D. Kim A. S. (2012b). Cost-effectiveness of dabigatran compared with warfarin for stroke prevention in patients with atrial fibrillation and prior stroke or transient ischemic attack. Stroke 43 (3), 881–883. 10.1161/STROKEAHA.111.641027 22308255

[B48] Kansal A. R. Sharma M. Bradley-Kennedy C. Clemens A. Monz B. U. Peng S. (2017). Dabigatran versus rivaroxaban for the prevention of stroke and systemic embolism in atrial fibrillation in Canada. Comparative efficacy and cost-effectiveness. Thromb. Haemost. 108 (10), 672–682. 10.1160/TH12-06-0388 22898892

[B49] Kansal A. R. Sorensen S. V. Gani R. Robinson P. Pan F. Plumb J. M. (2012). Cost-effectiveness of dabigatran etexilate for the prevention of stroke and systemic embolism in UK patients with atrial fibrillation. Heart 98 (7), 573–578. 10.1136/heartjnl-2011-300646 22422743 PMC3308473

[B50] Kim H. Kim H. Cho S. K. Kim J. B. Joung B. Kim C. (2019). Cost-effectiveness of rivaroxaban compared to warfarin for stroke prevention in atrial fibrillation. Korean Circ. J. 49 (3), 252–263. 10.4070/kcj.2018.0220 30468041 PMC6393322

[B51] Kleintjens J. Li X. Simoens S. Thijs V. Goethals M. Rietzschel E. R. (2013). Cost-effectiveness of rivaroxaban versus warfarin for stroke prevention in atrial fibrillation in the Belgian healthcare setting. Pharmacoeconomics 31 (10), 909–918. 10.1007/s40273-013-0087-9 24030788 PMC3824571

[B52] Kongnakorn T. Lanitis T. Annemans L. Thijs V. Goethals M. Marbaix S. (2015). Stroke and systemic embolism prevention in patients with atrial fibrillation in Belgium: comparative cost effectiveness of new oral anticoagulants and warfarin. Clin. Drug Invest 35 (2), 109–119. 10.1007/s40261-014-0253-7 25511639

[B53] Kourlaba G. Maniadakis N. Andrikopoulos G. Vardas P. (2014). Economic evaluation of rivaroxaban in stroke prevention for patients with atrial fibrillation in Greece. Cost. Eff. Resour. Alloc. 12 (1), 5. 10.1186/1478-7547-12-5 24512351 PMC3942277

[B54] Krejczy M. Harenberg J. Marx S. Obermann K. Frölich L. Wehling M. (2014). Comparison of cost-effectiveness of anticoagulation with dabigatran, rivaroxaban and apixaban in patients with non-valvular atrial fibrillation across countries. J. Thromb. Thrombolys 37 (4), 507–523. 10.1007/s11239-013-0989-6 24221805

[B55] Krejczy M. Harenberg J. Wehling M. Obermann K. Lip G. Y. (2015). Cost-effectiveness of anticoagulation in patients with nonvalvular atrial fibrillation with edoxaban compared to warfarin in Germany. Biomed. Res. Int. 2015, 876923. 10.1155/2015/876923 25853142 PMC4380099

[B56] Lanas F. Castro C. Vallejos C. Bustos L. de La Puente C. Velasquez M. (2017). Latin American clinical epidemiology network series - paper 2: apixaban was cost-effective vs. acenocoumarol in patients with nonvalvular atrial fibrillation with moderate to severe risk of embolism in Chile. J. Clin. Epidemiol. 86, 75–83. 10.1016/j.jclinepi.2016.05.018 27756577

[B57] Langkilde L. K. Bergholdt A. M. Overgaard M. (2012). Cost-effectiveness of dabigatran etexilate for stroke prevention in non-valvular atrial fibrillation. Applying RE-LY to clinical practice in Denmark. J. Med. Econ. 15 (4), 695–703. 10.3111/13696998.2012.673525 22397590

[B58] Lanitis T. Cotté F. E. Gaudin A. F. Kachaner I. Kongnakorn T. Durand-Zaleski I. (2014). Stroke prevention in patients with atrial fibrillation in France: comparative cost-effectiveness of new oral anticoagulants (apixaban, dabigatran, and rivaroxaban), warfarin, and aspirin. J. Med. Econ. 17 (8), 587–598. 10.3111/13696998.2014.923891 24831811

[B59] Lee S. Anglade M. Pisacane R. Pham D. Kluger J. Coleman C. (2012a). Cost-effectiveness of rivaroxaban compared to warfarin for stroke prophylaxis in atrial fibrillation. J. Am. Coll. Cardiol. 59 (13), E600. 10.1016/S0735-1097(12)60601-9 22651881

[B60] Lee S. Mullin R. Blazawski J. Coleman C. I. (2012b). Cost-effectiveness of apixaban compared with warfarin for stroke prevention in atrial fibrillation. Plos One 7 (10), e47473. 10.1371/journal.pone.0047473 23056642 PMC3467203

[B61] Lekuona I. Anguita M. Zamorano J. L. Rodríguez J. M. Barja De Soroa P. Pérez-Alcántara F. (2019). Would the use of Edoxaban be cost-effective for the prevention of stroke and systemic embolism in patients with nonvalvular atrial fibrillation in Spain? Rev. Española Cardiol. English Ed. 72 (5), 398–406. 10.1016/j.rec.2018.03.024 31007166

[B62] Li X. Tse V. C. Lau W. C. Cheung B. M. Lip G. Y. Wong I. C. (2016). Cost-effectiveness of apixaban versus warfarin in Chinese patients with non-valvular atrial fibrillation: a real-life and modelling analyses. Plos One 11 (6), e0157129. 10.1371/journal.pone.0157129 27362421 PMC4928891

[B63] Liao C. T. Lee M. C. Chen Z. C. Ku L. E. Wang J. D. Toh H. S. (2020). Cost-effectiveness analysis of oral anticoagulants in stroke prevention among patients with atrial fibrillation in Taiwan. Acta Cardiol. Sin. 36 (1), 50–61. 10.6515/ACS.202001_36(1).20190511A 31903008 PMC6933494

[B64] Lippi G. Sanchis-Gomar F. Cervellin G. (2021). Global epidemiology of atrial fibrillation: an increasing epidemic and public health challenge. Int. J. Stroke 16 (2), 217–221. 10.1177/1747493019897870 31955707

[B65] Liu C. Chen H. (2017). Cost-effectiveness analysis of apixaban, dabigatran, rivaroxaban, and warfarin for stroke prevention in atrial fibrillation in Taiwan. Clin. Drug Invest 37 (3), 285–293. 10.1007/s40261-016-0487-7 27988835

[B66] Liu L. Hong D. Ma K. Lu X. (2021). Cost-effectiveness of rivaroxaban versus warfarin in non-valvular atrial fibrillation patients with chronic kidney disease in China. J. Clin. Pharm. Ther. 46 (3), 658–668. 10.1111/jcpt.13318 33226144

[B67] López-López J. A. Sterne J. A. C. Thom H. H. Z. Higgins J. P. T. Hingorani A. D. Okoli G. N. (2017). Oral anticoagulants for prevention of stroke in atrial fibrillation: systematic review, network meta-analysis, and cost effectiveness analysis. BMJ 359, j5058. 10.1136/bmj.j5058 29183961 PMC5704695

[B68] Lorenzoni V. Pirri S. Turchetti G. (2021). Cost-effectiveness of direct non-vitamin K oral anticoagulants versus vitamin K antagonists for the management of patients with non-valvular atrial fibrillation based on available real-world evidence: the Italian national health system perspective. Clin. Drug Investig. 41 (3), 255–267. 10.1007/s40261-021-01002-z PMC7946694 33587284

[B69] Lv P. Yang L. (2019). Cost-utility analysis of left atrial appendage closure and new oral anticoagulants for stroke prevention in patients with atrial fibrillation. China J. Pharm. Econ. 14 (03), 15–23. 10.12010/j.issn.1673-5846.2019.03.003

[B70] Magnuson E. A. Vilain K. Wang K. Li H. Kwong W. J. Antman E. M. (2015). Cost-effectiveness of edoxaban vs warfarin in patients with atrial fibrillation based on results of the ENGAGE AF-TIMI 48 trial. Am. Heart J. 170 (6), 1140–1150. 10.1016/j.ahj.2015.09.011 26678636

[B71] Mendoza J. A. Silva F. A. Rangel L. M. (2019). Cost-effectiveness of new oral anticoagulants and warfarin in atrial fibrillation from adverse events perspective. Rev. Colomb. Cardiol. 26 (2), 70–77. 10.1016/j.rccar.2018.10.011

[B72] Mensch A. Stock S. Stollenwerk B. Müller D. (2015). Cost effectiveness of rivaroxaban for stroke prevention in German patients with atrial fibrillation. Pharmacoeconomics 33 (3), 271–283. 10.1007/s40273-014-0236-9 25404426

[B73] Moher D. Liberati A. Tetzlaff J. Altman D. G. PRISMA Group (2009). Preferred reporting items for systematic reviews and meta-analyses: the PRISMA statement. Plos Med. 6 (7), e1000097. 10.1371/journal.pmed.1000097 19621072 PMC2707599

[B74] Morais J. Aguiar C. McLeod E. Chatzitheofilou I. Fonseca S. I. Pereira S. (2014). Cost-effectiveness of rivaroxaban for stroke prevention in atrial fibrillation in the Portuguese setting. Rev. Port. Cardiol. 33 (9), 535–544. 10.1016/j.repc.2014.02.020 25241380

[B75] Ng S. S. Nathisuwan S. Phrommintikul A. Chaiyakunapruk N. (2020). Cost-effectiveness of warfarin care bundles and novel oral anticoagulants for stroke prevention in patients with atrial fibrillation in Thailand. Thromb. Res. 185, 63–71. 10.1016/j.thromres.2019.11.012 31770689

[B76] Nguyen E. Egri F. Mearns E. S. White C. M. Coleman ci (2016). Cost-effectiveness of high-dose edoxaban compared with adjusted-dose warfarin for stroke prevention in non-valvular atrial fibrillation patients. Pharmacotherapy 36 (5), 488–495. 10.1002/phar.1746 27015873

[B77] Noviyani R. Youngkong S. Nathisuwan S. Bagepally B. S. Chaikledkaew U. Chaiyakunapruk N. (2022). Economic evaluation of direct oral anticoagulants (DOACs) versus vitamin K antagonists (VKAs) for stroke prevention in patients with atrial fibrillation: a systematic review and meta-analysis. BMJ Evidence-Based Med. 27 (4), 215–223. 10.1136/bmjebm-2020-111634 PMC9340051 34635480

[B78] Nshimyumukiza L. Duplantie J. Gagnon M. Douville X. Fournier D. Lindsay C. (2013). Dabigatran versus warfarin under standard or pharmacogenetic-guided management for the prevention of stroke and systemic thromboembolism in patients with atrial fibrillation: a cost/utility analysis using an analytic decision model. Thromb. J. 11 (1), 14. 10.1186/1477-9560-11-14 23866305 PMC3765702

[B79] Patel M. R. Mahaffey K. W. Garg J. Pan G. Singer D. E. Hacke W. (2011). Rivaroxaban versus warfarin in nonvalvular atrial fibrillation. New Engl. J. Med. 365 (10), 883–891. 10.1056/NEJMoa1009638 21830957

[B80] Pepe RDSC Bolzachini S. N. Gomes D. M. T. Jansen DOFM Da C. D. F. Soares P. L. (2015). Cost-effectiveness and cost-utility analyses of dabigatran compared with warfarin in patients with nonvalvular atrial fibrillation and risk factors for stroke and systemic embolism within Brazilian private and public health care systems perspectives. Value Health Reg. Issues 8, 36–42. 10.1016/j.vhri.2015.02.003 29698169

[B81] Pink J. Pirmohamed M. Lane S. Hughes D. A. (2014). Cost-effectiveness of pharmacogenetics-guided warfarin therapy vs. alternative anticoagulation in atrial fibrillation. Clin. Pharmacol. Ther. 95 (2), 199–207. 10.1038/clpt.2013.190 24067746

[B82] Pletscher M. Plessow R. Eichler K. Wieser S. (2013). Cost-effectiveness of dabigatran for stroke prevention in atrial fibrillation in Switzerland. Swiss Med. Wkly. 143, w13732. 10.4414/smw.2013.13732 23300013

[B83] Rattanachotphanit T. Limwattananon C. Waleekhachonloet O. Limwattananon P. Sawanyawisuth K. (2019). Cost-effectiveness analysis of direct-acting oral anticoagulants for stroke prevention in Thai patients with non-valvular atrial fibrillation and a high risk of bleeding. Pharmacoeconomics 37 (2), 279–289. 10.1007/s40273-018-0741-3 30387074

[B84] Raunbak S. M. Sørensen A. S. Hansen L. Skjøth F. Larsen T. B. Ehlers L. H. (2022). Cost effectiveness of patient self-managed warfarin compared with direct oral anticoagulants in atrial fibrillation: an economic evaluation in a Danish healthcare sector setting. PharmacoEconomics - Open 6 (4), 483–494. 10.1007/s41669-022-00337-3 35665481 PMC9283633

[B85] Rivolo S. Di Fusco M. Polanco C. Kang A. Dhanda D. Savone M. (2021). Cost-effectiveness analysis of apixaban versus vitamin K antagonists for antithrombotic therapy in patients with atrial fibrillation after acute coronary syndrome or percutaneous coronary intervention in Spain. Plos One 16 (11), e0259251. 10.1371/journal.pone.0259251 34767564 PMC8589164

[B86] Rognoni C. Marchetti M. Quaglini S. Liberato N. L. (2014). Apixaban, dabigatran, and rivaroxaban versus warfarin for stroke prevention in non-valvular atrial fibrillation: a cost-effectiveness analysis. Clin. Drug Invest. 34 (1), 9–17. 10.1007/s40261-013-0144-3 24135964

[B87] Rognoni C. Marchetti M. Quaglini S. Liberato N. L. (2015). Edoxaban versus warfarin for stroke prevention in non-valvular atrial fibrillation: a cost-effectiveness analysis. J. Thromb. Thrombolysis 39 (2), 149–154. 10.1007/s11239-014-1104-3 24973057

[B88] Roth G. A. Mensah G. A. Johnson C. O. Addolorato G. Ammirati E. Baddour L. M. (2020). Global burden of cardiovascular diseases and risk factors, 1990-2019: update from the GBD 2019 study. J. Am. Coll. Cardiol. 76 (25), 2982–3021. 10.1016/j.jacc.2020.11.010 33309175 PMC7755038

[B89] Salata B. M. Hutton D. W. Levine D. A. Froehlich J. B. Barnes G. D. (2016). Cost-effectiveness of dabigatran (150 mg twice daily) and warfarin in patients ≥ 65 Years with nonvalvular atrial fibrillation. Am. J. Cardiol. 117 (1), 54–60. 10.1016/j.amjcard.2015.09.048 26552509 PMC4780215

[B90] Salcedo J. Hay J. W. Lam J. (2019). Cost-effectiveness of rivaroxaban versus warfarin for treatment of nonvalvular atrial fibrillation in patients with worsening renal function. Int. J. Cardiol. 282, 53–58. 10.1016/j.ijcard.2018.11.087 30518479

[B91] Shah A. Shewale A. Hayes C. J. Martin B. C. (2016). Cost-effectiveness of oral anticoagulants for ischemic stroke prophylaxis among nonvalvular atrial fibrillation patients. Stroke 47 (6), 1555–1561. 10.1161/STROKEAHA.115.012325 27103018

[B92] Shah S. V. Gage B. F. (2011). Cost-effectiveness of dabigatran for stroke prophylaxis in atrial fibrillation. Circulation 123 (22), 2562–2570. 10.1161/CIRCULATIONAHA.110.985655 21606397

[B93] Sorensen S. V. Kansal A. R. Connolly S. Peng S. Linnehan J. Bradley-Kennedy C. (2011). Cost-effectiveness of dabigatran etexilate for the prevention of stroke and systemic embolism in atrial fibrillation: a Canadian payer perspective. Thromb. Haemost. 105 (5), 908–919. 10.1160/TH11-02-0089 21431243

[B94] Steffel J. Collins R. Antz M. Cornu P. Desteghe L. Haeusler K. G. (2021). 2021 European heart rhythm association practical guide on the use of non-vitamin K antagonist oral anticoagulants in patients with atrial fibrillation. EP Eur. 23 (10), 1612–1676. 10.1093/europace/euab065 PMC11636576 33895845

[B95] Stevanovic J. Pompen M. Le H. H. Rozenbaum M. H. Tieleman R. G. Postma M. J. (2014). Economic evaluation of apixaban for the prevention of stroke in non-valvular atrial fibrillation in The Netherlands. Plos One 9 (8), e103974. 10.1371/journal.pone.0103974 25093723 PMC4122386

[B96] Thom H. Hollingworth W. Sofat R. Wang Z. Fang W. Bodalia P. N. (2019). Directly acting oral anticoagulants for the prevention of stroke in atrial fibrillation in England and Wales: cost-effectiveness model and value of information analysis. MDM Policy Pract. 4 (2), 2381468319866828. 10.1177/2381468319866828 31453363 PMC6699015

[B97] van Hulst M. Stevanovic J. Jacobs M. S. Tieleman R. G. Kappelhoff B. Postma M. J. (2018). The cost-effectiveness and monetary benefits of dabigatran in the prevention of arterial thromboembolism for patients with non-valvular atrial fibrillation in The Netherlands. J. Med. Econ. 21 (1), 38–46. 10.1080/13696998.2017.1372222 28836865

[B98] Vargas E. R. Sposato L. A. Lee S. A. W. Hachinski V. Cipriano L. E. (2018). Anticoagulation therapy for atrial fibrillation in patients with Alzheimer's disease. Stroke 49 (12), 2844–2850. 10.1161/STROKEAHA.118.022596 30571418

[B99] Verhoef T. I. Redekop W. K. Hasrat F. de Boer A. Maitland-van Der Zee A. H. (2014). Cost effectiveness of new oral anticoagulants for stroke prevention in patients with atrial fibrillation in two different European healthcare settings. Am. J. Cardiovasc Drug 14 (6), 451–462. 10.1007/s40256-014-0092-1 PMC4250561 25326294

[B100] Vilain K. A. Yang M. C. Hui T. E. Wang K. Li H. Hsu W. H. (2017). Cost-effectiveness of Edoxaban vs. warfarin in patients with atrial fibrillation based on results of the ENGAGE AF - TIMI 48 trial: Taiwanese perspective. Value Health Reg. Issues 12, 74–83. 10.1016/j.vhri.2017.03.011 28648320

[B101] Walter E. Voit M. Eichhober G. (2021). Cost-effectiveness analysis of apixaban compared to other direct oral anticoagulants for prevention of stroke in Austrian atrial fibrillation patients. Expert Rev. Pharm. Out. 21 (2), 265–275. 10.1080/14737167.2020.1798233 32700584

[B102] Wan Y. Chen J. L. Lu Z. G. Chen F. (2014). Cost-effectiveness of dabigatran compared with warfarin for stroke prevention in atrial fibrillation in China. Chin. J. Health Statistics 31 (04), 608–611.

[B103] Wang C. Pham P. N. Thai T. N. Brown J. D. (2020). Updating the cost effectiveness of oral anticoagulants for patients with atrial fibrillation based on varying stroke and bleed risk profiles. Pharmacoeconomics 38 (12), 1333–1343. 10.1007/s40273-020-00960-0 32924092

[B104] Wang S. H. Huang C. Qi L. M. (2022). Pharmacoeconomic valuation of strategies for treatment of nonvalvular atrial fibrillation based on Markov model. China J. Pharm. Econ. 17 (08), 39–44. 10.12010/j.issn.1673-5846.2022.08.006

[B105] Wang Y. Xie F. Kong M. C. Lee L. H. Ng H. J. Ko Y. (2014). Cost-effectiveness of dabigatran and rivaroxaban compared with warfarin for stroke prevention in patients with atrial fibrillation. Cardiovasc Drugs Ther. 28 (6), 575–585. 10.1007/s10557-014-6558-1 25319314

[B106] Wei H. Cui C. Cui X. Liu Y. Li D. (2021). Cost-effectiveness analysis of dabigatran, rivaroxaban and warfarin in the prevention of stroke in patients with atrial fibrillation in China. Bmc Health Serv. Res. 21 (1), 96. 10.1186/s12913-021-06084-1 33509171 PMC7841891

[B107] Wisløff T. Hagen G. Klemp M. (2014). Economic evaluation of warfarin, dabigatran, rivaroxaban, and apixaban for stroke prevention in atrial fibrillation. Pharmacoeconomics 32 (6), 601–612. 10.1007/s40273-014-0152-z 24715603 PMC4031399

[B108] Wouters H. Thijs V. Annemans L. (2013). Cost-effectiveness of dabigatran etexilate in the prevention of stroke and systemic embolism in patients with atrial fibrillation in Belgium. J. Med. Econ. 16 (3), 407–414. 10.3111/13696998.2013.766200 23320796

[B109] Wu B. Kun L. Liu X. He B. (2014). Cost-effectiveness of different strategies for stroke prevention in patients with atrial fibrillation in a health resource-limited setting. Cardiovasc Drugs Ther. 28 (1), 87–98. 10.1007/s10557-013-6490-9 24048510

[B110] Wu Y. Feng J. Peng Y. Rong P. P. Li M. Zhou B. H. (2016a). Cost-effectiveness of novel oral anticoagulants versus Warfarin for stroke prevention in patients with non-valvular atrial fibrillation. Chin. Hosp. Pharm. J. 36 (12), 1003–1007. 10.13286/j.cnki.chinhosppharmacyj.2016.12.11

[B111] Wu Y. Feng J. Peng Y. Rong P. P. Li M. Zhou B. H. (2016b). Cost-effectiveness of apixaban vs warfarin for secondary stroke prevention of atrial fibrillation. Chin. J. Mod. Appl. Pharm. 33 (09), 1183–1188. 10.13748/j.cnki.issn1007-7693.2016.09.021

[B112] Wu Y. Zhang C. Gu Z. C. (2021). Cost-effectiveness analysis of direct oral anticoagulants vs. vitamin K antagonists in the elderly with atrial fibrillation: insights from the evidence in a real-world setting. Front. Cardiovasc Med. 8, 675200. 10.3389/fcvm.2021.675200 34268343 PMC8275875

[B113] Xie F. Zhou T. (2022). Industry sponsorship bias in cost effectiveness analysis: registry based analysis. BMJ 377, e069573. 10.1136/bmj-2021-069573 35732297 PMC9214880

[B114] Yang Z. Y. (2021). The clinical evaluation of warfarin and its pharmacoeconomic evaluation with rivaroxaban. Chengdu, China: A Master Thesis Submitted to University of Electronic Science and Technology of China.

[B115] You J. H. S. (2014). Novel oral anticoagulants versus warfarin therapy at various levels of anticoagulation control in atrial fibrillation—a cost-effectiveness analysis. J. Gen. Intern Med. 29 (3), 438–446. 10.1007/s11606-013-2639-2 24132628 PMC3930767

[B116] Zhao Y. J. Lin L. Zhou H. J. Tan K. T. Chew A. P. Foo C. G. (2016). Cost-effectiveness modelling of novel oral anticoagulants incorporating real-world elderly patients with atrial fibrillation. Int. J. Cardiol. 220, 794–801. 10.1016/j.ijcard.2016.06.087 27400183

[B117] Zheng Y. Sorensen S. V. Gonschior A. Noack H. Heinrich-Nols J. Sunderland T. (2014). Comparison of the cost-effectiveness of new oral anticoagulants for the prevention of stroke and systemic embolism in atrial fibrillation in a UK setting. Clin. Ther. 36 (12), 2015–2028. 10.1016/j.clinthera.2014.09.015 25438722

[B118] Zhou H. Nie X. Jiang M. Dong W. (2022). Cost-effectiveness of anticoagulants for preventing stroke in patients with non-valvular atrial fibrillation in mainland China. J. Clin. Pharm. Ther. 47 (4), 523–530. 10.1111/jcpt.13575 34783090

