# Supplementary material for: Factors influencing the cost-effectiveness of novel oral anticoagulants compared to vitamin K antagonists in patients with atrial fibrillation: a systematic review
Source: Front Pharmacol. 2025 Mar 28;16:1441754. doi: 10.3389/fphar.2025.1441754 (PMC11986374; doi:10.3389/fphar.2025.1441754)
Supplement: Supplementary file 1 [file DataSheet1.docx]

**Supplementary Materials**

# Searched Details from Databases

## Supplementary Table 1 Searched Details from Pubmed

**Table 1 Searched Details from Pubmed**

| **Database** | **Search strategy** |
| --- | --- |
| **PubMed** | 1 atrial fibrillation[ti,ab]  2 atrial fibrillation[Mesh]  3 1 OR 2  4 noac* OR oral anticoagulant* OR non vitamin K antagonist*[ti,ab]  5 apixaban OR rivaroxaban OR dabigatran OR edoxaban[ti,ab]  6 4 OR 5  7 economic evaluation OR economic analysis OR pharmaco economic OR pharmacoeconomi*[ti,ab]  8 cost effectiveness OR cost utility OR cost benefit OR cost minimization OR cost minimisation[ti,ab]  9 7 OR 8  10 3 AND 6 AND 9 |

## Supplementary Table 2 Searched Details from Web of Science

**Table 2 Searched Details from** **Web of Science**

| **Database** | **Search strategy** |
| --- | --- |
| **Web of Science** | 1 TS=(atrial fibrillation)  2 TS=(noac* OR oral anticoagulant* OR non vitamin K antagonist*)  3 TS=(apixaban OR rivaroxaban OR dabigatran OR edoxaban)  4 2 OR 3  5 TS=(economic evaluation OR economic analysis OR pharmaco economic OR pharmacoeconomi*)  6 TS=(cost effectiveness OR cost utility OR cost benefit OR cost minimization OR cost minimisation)  7 5 OR 6  8 1 AND 4 AND 7 |

## Supplementary Table 3 Searched Details from the Cochrane Library

**Table 3 Searched Details from the Cochrane Library**

| **Database** | **Search strategy** |
| --- | --- |
| **The Cochrane Library** | 1 (atrial fibrillation):ti,ab,kw  2 (noac* OR oral anticoagulant* OR non vitamin K antagonist*):ti,ab,kw  3 (apixaban OR rivaroxaban OR dabigatran OR edoxaban):ti,ab,kw  4 2 OR 3  5 (economic evaluation OR economic analysis OR pharmaco economic OR pharmacoeconomi*):ti,ab,kw  6 (cost effectiveness OR cost utility OR cost benefit OR cost minimization OR cost minimisation):ti,ab,kw  7 5 OR 6  8 1 AND 4 AND 7 |

## Supplementary Table 4 Searched Details from Embase

**Table 4 Searched Details from Embase**

| **Database** | **Search strategy** |
| --- | --- |
| **Embase** | 1 'atrial fibrillation'/exp  2 (‘noac*’ OR ‘oral anticoagulant*’ OR ‘non vitamin K antagonist*’):ti,ab,kw  3 (‘apixaban’ OR ‘rivaroxaban’ OR ‘dabigatran’ OR ‘edoxaban’):ti,ab,kw  4 2 OR 3  5 (‘economic evaluation’ OR ‘economic analysis’ OR ‘pharmaco economic’ OR ‘pharmacoeconomi*’):ti,ab,kw  6 (‘cost effectiveness’ OR ‘cost utility’ OR ‘cost benefit’ OR ‘cost minimization’ OR ‘cost minimisation’):ti,ab,kw  7 5 OR 6  8 1 AND 4 AND 7 |

## Supplementary Table 5 Searched Details from China National Knowledge Infrastructure (CNKI)

**Table 5 Searched Details from CNKI**

| **Database** | **Search strategy** |
| --- | --- |
| **CNKI** | 1 TKA=(房颤+心房颤动+心房纤颤)  2 TKA=(经济评价+经济评估+经济研究+药物经济学+马尔科夫模型+决策模型+增量分析)  3 TKA=(成本结果+’成本-结果’+成本效果+’成本-效果’+成本效用+’成本-效用’+成本效益+’成本-效益’+最小成本分析)  4 2 OR 3  5 TKA=(抗凝药+利伐沙班+达比加群酯+依度沙班+艾多沙班+阿哌沙班+非维生素K拮抗剂)  6 1 AND 4 AND 5 |

## Supplementary Table 6 Searched Details from VIP Database

**Table 6 Searched Details from VIP Database**

| **Database** | **Search strategy** |
| --- | --- |
| **VIP**  **Database** | 1 主题=(房颤OR心房颤动OR心房纤颤)  2 主题=(经济评价OR经济评估OR经济研究OR药物经济学OR马尔科夫模型OR决策模型OR增量分析)  3 主题=(成本结果OR’成本-结果’OR成本效果OR’成本-效果’OR成本效用OR’成本-效用’OR成本效益OR’成本-效益’OR最小成本分析)  4 2 OR 3  5 主题=(抗凝药OR利伐沙班OR达比加群酯OR依度沙班OR艾多沙班OR阿哌沙班OR非维生素K拮抗剂)  6 1 AND 4 AND 5 |

## Supplementary Table 7 Searched Details from Wanfang Data

**Table 7 Searched Details from Wanfang Data**

| **Database** | **Search strategy** |
| --- | --- |
| **Wanfang**  **Data** | 1 主题：(房颤OR心房颤动OR心房纤颤)  2 主题：(经济评价OR经济评估OR经济研究OR药物经济学OR马尔科夫模型OR决策模型OR增量分析)  3 主题：(成本结果OR’成本-结果’OR成本效果OR’成本-效果’OR成本效用OR’成本-效用’OR成本效益OR’成本-效益’OR最小成本分析)  4 2 OR 3  5 主题：(抗凝药OR利伐沙班OR达比加群酯OR依度沙班OR艾多沙班OR阿哌沙班OR非维生素K拮抗剂)  6 1 AND 4 AND 5 |

#
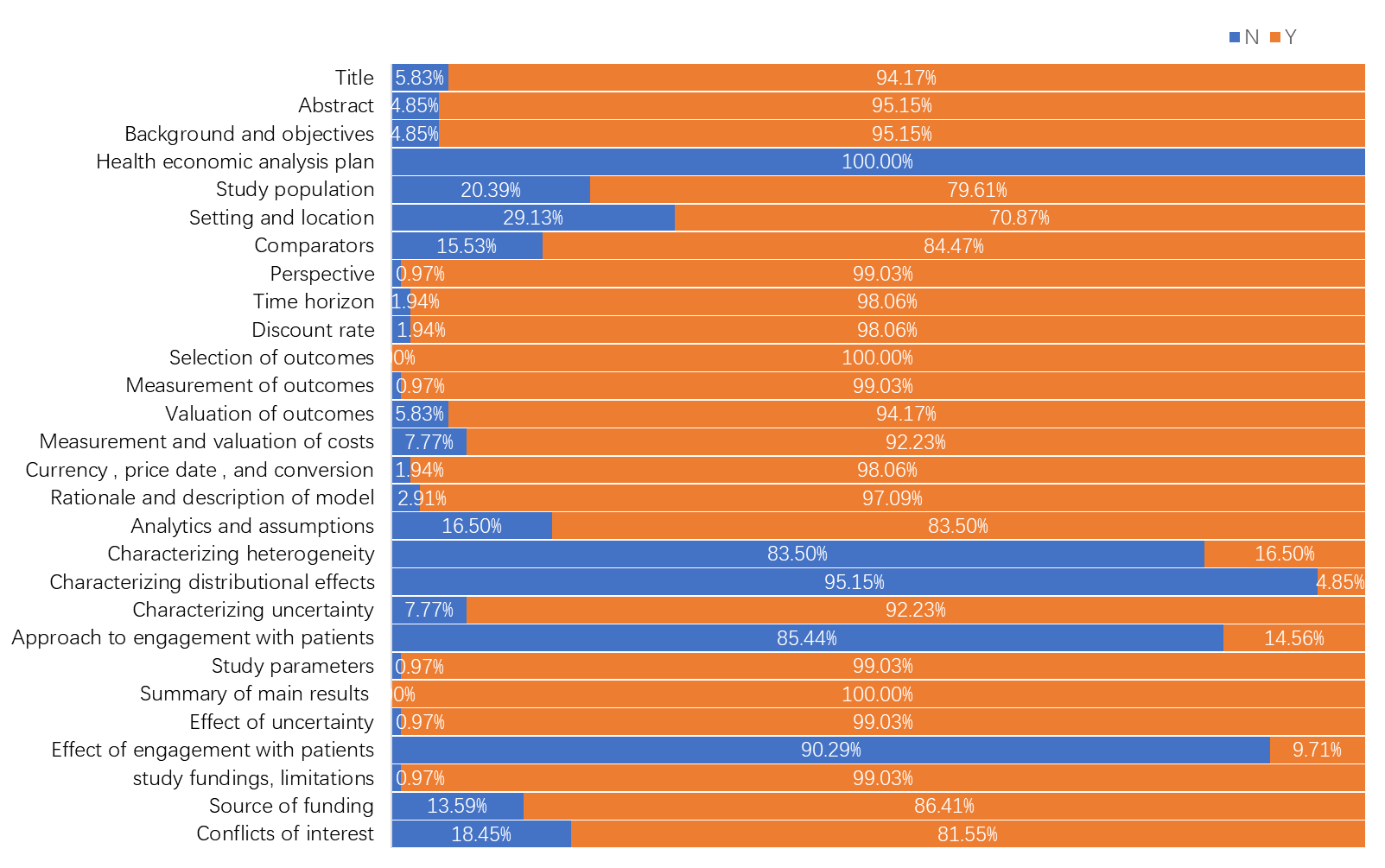
A summary figure of the quality assessment

**Figure 1 Statistics of CHEERS 2022 checklist of included studies**
